# Supplementary material for: A Critical View of the Application of the APEX Software (Aqueous Photochemistry of Environmentally-Occurring Xenobiotics) to Predict Photoreaction Kinetics in Surface Freshwaters
Source: Molecules. 2019 Dec 18;25(1):9. doi: 10.3390/molecules25010009 (PMC7017383; doi:10.3390/molecules25010009)
Supplement: Supplementary file 1 [file molecules-25-00009-s001.zip › 3.2.4_gcc-4.4.0.zip!/3.2.4_gcc-4.4.0/3.2.4_gcc-4.4.0/MSYS/doc/msys/MSYS_LICENSE.rtf]

Minimal SYStem
Version 1.0.11
http://www.mingw.org/wiki/msys/

License, Use and Redistribution

MSYS contains several different other packages.  Most of those packages are licensed by the GNU Public License (GPL). 

You may use MSYS on any number of systems.  There is no restriction to your use.  You may use MSYS commercially as well as privately.  You the user assume the responsibility for the use of the files, binary or text, and there is no guarantee or warranty, expressed or implied, including but not limited to the implied warranties of merchantability and fitness for a particular purpose.   You assume all responsibility and agree to hold no entity, copyright holder or distributors liable for any loss of data or inaccurate representations of data as a result of using MSYS.

You may redistribute MSYS in part or in whole as long as you follow the guidelines of redistribution of each license contained within.  To be certain that you are being legally compliant, always distribute the source.  Distribution of source is your responsibility should you decide to redistribute MSYS.  If you distribute MSYS via a web site then you must put a copy of the source for that version of MSYS on your web site as well.  If you distribute MSYS via removable media then you must distribute that version of MSYS source with that same type of removable media.

Binaries created from the use of MSYS and of MinGW are not bound by any license found within this package unless you use a library that is itself covered by the GPL license.  If you wish to create proprietary software then don't use libiberty.a or any other GPL licensed library.  A library licensed with LGPL (Lesser GPL) may be used by proprietary software without GPL infection as special permission within the LGPL has given you this right.

Earnie.
Earnie@users.sf.net
